# The predictive value of the Singh index for the risk of InterTAN intramedullary fixation failure in elderly patients with intertrochanteric fractures

**DOI:** 10.1186/s12891-022-05741-8

**Published:** 2022-08-12

**Authors:** Zhe Xu, Guang Tian, Chen Liu, Yangjiang Xie, Ruguo Zhang

**Affiliations:** 1 Department of Orthopaedics, General Medical 300 Hospital, Guiyang, 550004 China; 2 National-Local Joint Engineering Laboratory of Cell Engineering and Biomedicine, Guiyang, 550004 China

**Keywords:** Singh index, Elderly patients, Intertrochanteric fracture, Internal fixation failure

## Abstract

**Background:**

To investigate the predictive value of the Singh index for the risk of InterTAN intramedullary fixation failure in elderly patients with intertrochanteric fracture to guide clinical treatment.

**Methods:**

A total of 360 patients were divided into the Singh (I ~ II) (*n* = 120), Singh (III ~ IV) (*n* = 120) and Singh (V ~ VI) (*n* = 120) groups. Visual analog scale (VAS) and Harris scores were recorded at 1, 6, 12, 18 and 24 months after the operation. The correlation between the Singh index and the T-score of the total hip and femoral neck was analyzed. Logistic regression was used to analyze the relationship between the Singh index and internal fixation failure; the types of internal fixation failure were also analyzed.

**Results:**

The Harris scores of the Singh (I ~ II) group were lower than those of the Singh (III ~ IV) and Singh (V ~ VI) groups 12, 18 and 24 months after surgery (*P* < 0.05). The Singh index was significantly correlated with the T-score of the total hip and femoral neck (*P* = 0.00, *r* = 0.89; *P* = 0.00, *r* = 0.83). The Singh (I ~ II) group had the lowest internal fixation survival rate within 24 months (*P* = 0.01). The Singh index was an independent predictor of internal fixation failure (*P* < 0.05). Lag screw cutting-out was the main type of internal fixation failure in the three groups (*P* = 0.00).

**Conclusion:**

The Singh index is significantly correlated with the bone mineral density of the femoral neck and total hip. The Singh (I ~ II) group had lower Harris scores and a lower internal fixation survival rate than the other two groups. The Singh index is an independent predictor of internal fixation failure, especially lag screw cutting-out, after InterTAN fixation.

**Supplementary Information:**

The online version contains supplementary material available at 10.1186/s12891-022-05741-8.

## Background

At present, there are a variety of methods used to treat intertrochanteric fractures in elderly patients, including the dynamic hip system (DHS), femoral intramedullary nails, hip replacement, etc. [1–4]. For unstable intertrochanteric fractures, intertrochanteric antegrade nail (InterTAN) fixation is the main method used. It has achieved satisfactory clinical results in some clinical studies [5–9]. Although some articles have proven that InterTAN has more advantages than other methods [2, 5]. internal fixation failure is a common complication [10]. Some studies have proposed the concept of tip-apex distances (TADs). Studies believe that a TAD < 25 mm can effectively reduce the cutting-out rate of the lag screw [11, 12]. In recent years, some studies have shown that the thickness of the lateral wall, satisfactory reduction and positive support of the medial wall can also affect the survival time of internal fixation [13–16].

Although continuous improvements in technical methods have reduced the incidence of internal fixation failure, the clinical incidence of intramedullary nail failure in elderly patients with intertrochanteric fracture is still high. For elderly patients with femoral intertrochanteric fracture, although some patients have an ideal TAD, lateral wall thickness and medial wall support and satisfactory reduction, internal fixation failure still occurs after surgery. The reason for this situation is not clear. This study speculates that the most likely factor is osteoporosis. Therefore, a simple, inexpensive and effective imaging-based evaluation method that can predict the risk of internal fixation failure is urgently needed in the clinic.

In 1978, Singh et al. [17] proposed the concept of the Singh index, which is a semiquantitative morphological index used to determine the loss of trabecular bone in the proximal femur by X-ray plain film. Singh divided trabecular bone into 6 grades according to the change in trabecular bone in the proximal femur, and the loss of trabecular bone gradually increased with decreasing grade. Some studies have shown that the Singh index has a significant correlation with the mechanical strength of the hip [18, 19]. Some studies have taken it as an important index for predicting hip fracture [20, 21], but few studies have used it as an imaging tool to predict internal fixation failure after InterTAN fixation.

The Singh index, as a semiquantitative index of bone trabecular density of the hip, may indirectly reflect the bone strength of the hip. This study hypothesized that the Singh index is an independent risk factor for InterTAN fixation failure and is related to survival time. It can be used as a simple and inexpensive imaging-based evaluation tool to guide the selection of appropriate clinical treatment.

## Materials and methods

All experimental protocols were approved by the Ethics Committee of General Medical 300 Hospital and informed consent was obtained from all patients. All methods were carried out in accordance with relevant guidelines and Declaration of the Helsinki. This study retrospectively analyzed 360 elderly patients with intertrochanteric fractures from 2015 to 2020. All patients were treated with InterTAN intramedullary nailing (Trigen, Smith & Nephew, London). None of the patients received regular bisphosphonate anti-osteoporosis treatment after the operation. All patients underwent preoperative pelvic plain film radiography, and the Singh index was calculated by an experienced imaging physician. Patients were divided into the Singh (I ~ II) (*n* = 120, Singh I = 65, Singh II = 55), Singh (III ~ IV) (*n* = 120, Singh III = 60, Singh IV = 60), and Singh (V ~ VI) (*n* = 120, Singh V = 70, Singh VI = 50) groups. According to the anteroposterior and lateral hip X-rays after surgery, the TAD, preoperative lateral wall thickness, and postoperative difference in cervical shaft angle of each group were statistically analyzed (Table 1).

**Table 1:** Preoperative profiles

Variables	Singh (I ~ II)	Singh (III ~ IV)	Singh (V ~ VI)	*P* value
Age (year)	84.72 ± 3.15	84.05 ± 3.59	83.80 ± 2.25	*P* = 0.38
Sex (male/female)	51/69	60/60	63/57	*P* = 0.65
Body mass index (BMI)	22.68 ± 2.85	23.13 ± 1.81	22.52 ± 2.54	*P* = 0.52
Affected side (Left/Right)	57/63	54/66	69/51	*P* = 0.50
**Jensen-Evans Type**				*P* = 0.98
II	7	9	8	
III	23	24	19	
IV	45	48	48	
V	45	39	45	
**OTA/AO classification**				
A1	10	15	14	
A2	68	56	60	
A3	42	49	46	
Lateral wall thickness	24.23 ± 0.95	24.05 ± 1.32	24.70 ± 1.81	*P* = 0.11
**Reduction effect**				
Tip-apex distance (TAD)	23.15 ± 1.59	23.28 ± 2.14	24.00 ± 2.49	*P* = 0.15
Difference in cervical shaft angle	4.40 ± 0.98	4.78 ± 1.19	4.50 ± 0.82	*P* = 0.23
**Basic diseases**				*P* = 0.58
Hypertension	12	18	21	
Type II diabetes	15	21	9	
Severe Cardiovascular disease	0	0	0	
Severe Endocrine disease	0	0	0	

### Inclusion and exclusion criteria

**Fig. 1:**
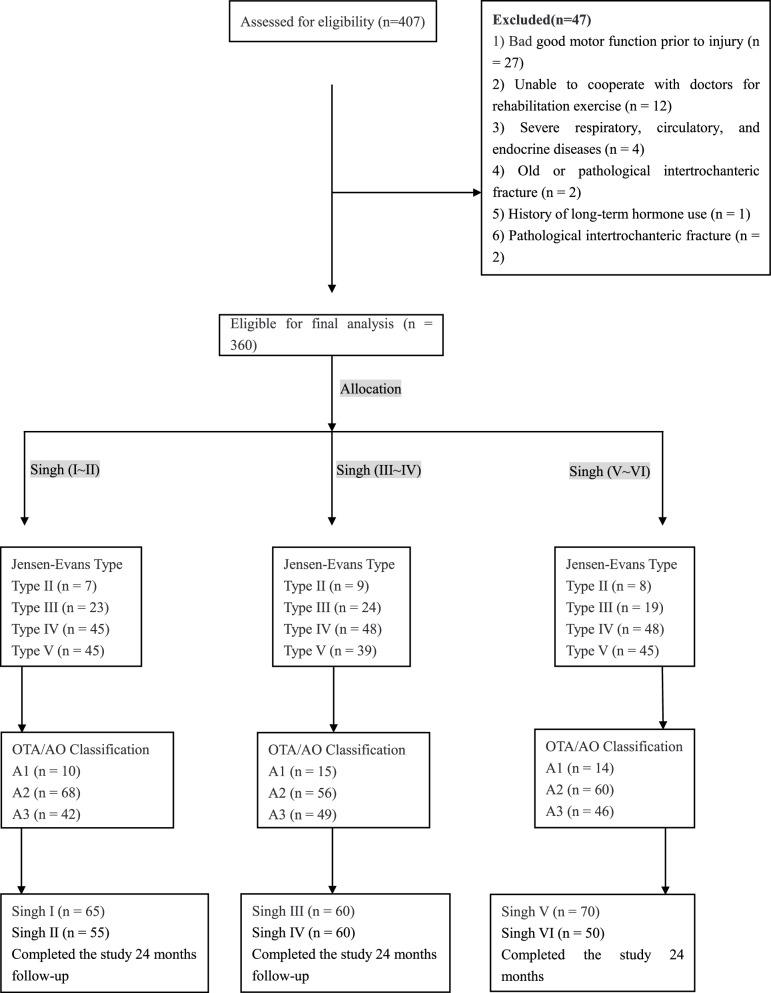
Flow diagram of the clinical trial

The inclusion criteria were as follows (Fig. 1): 1) aged 80–90 years; 2) good motor function prior to injury; 3) ability to cooperate with doctors for rehabilitation exercise; 4) bilateral hip joint X-ray availability; and 5) no history of long-term hormone use. The exclusion criteria were as follows: 1) multiple fractures; 2) old or pathological intertrochanteric fracture; 3) femoral neck fracture or subtrochanteric fracture; and 4) severe respiratory, circulatory, and endocrine diseases.

### The Singh index

**Fig. 2:**
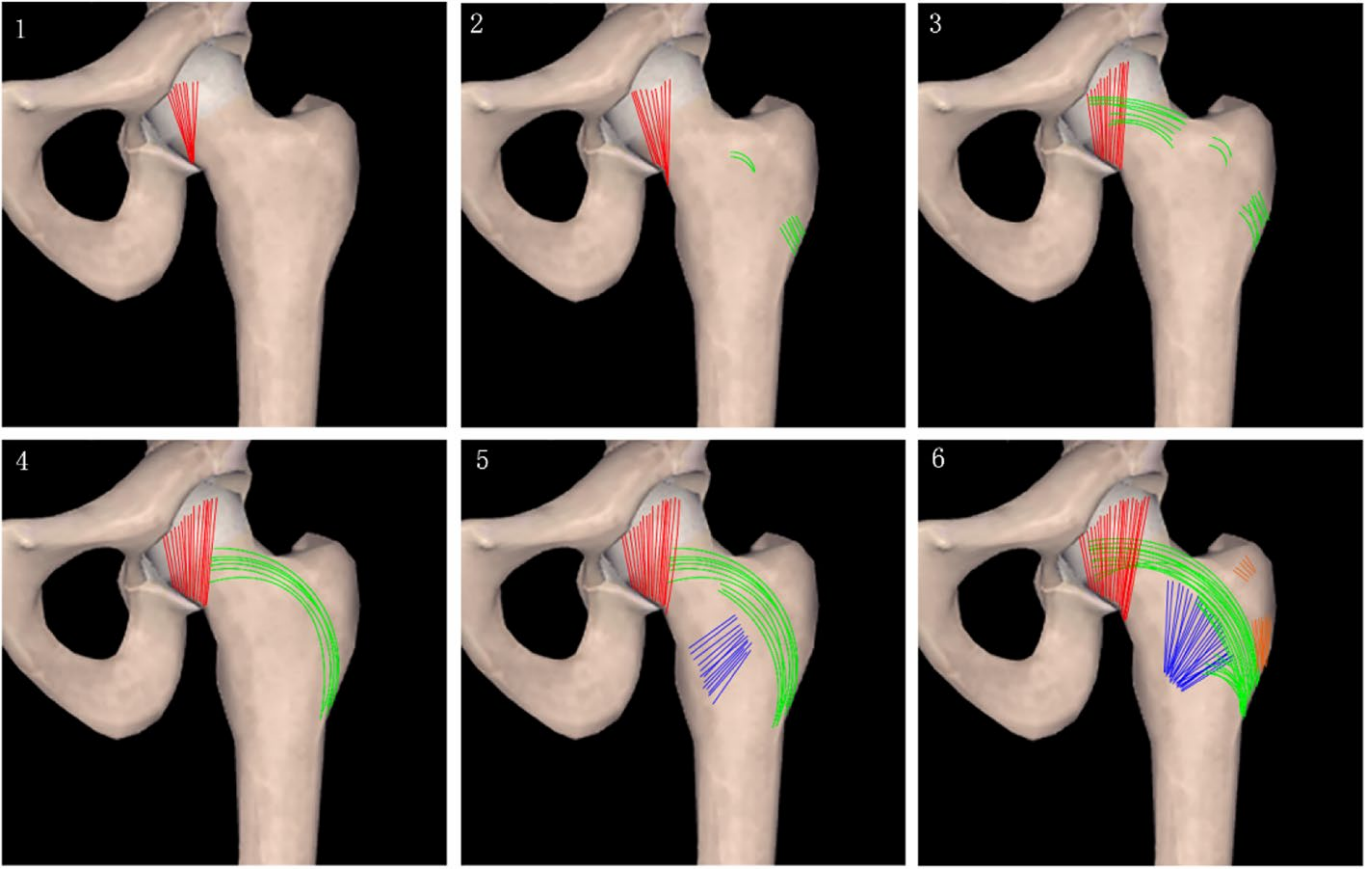
From Singh I to Singh VI, the distribution of bone trabecular density gradually increases

The Singh index is mainly used to evaluate the distribution of trabecular bone in the proximal femur of the nonfracture side on pelvic X-ray films, and it is usually divided into grades I ~ VI. With increasing grade, the distribution of trabecular bone density gradually increases (Fig. 2). It is difficult to distinguish adjacent Singh index grades in clinical practice [22, 23]. In this study, the grades were divided into three groups: I ~ II, III ~ IV, and V ~ VI. Inter- and intraobserver reliability (three radiologists, three traumatologists, and five patients) was evaluated by the intraclass correlation coefficient (ICC) value = 0.81, which had high measurement reliability (*P* = 0.00).

### Measurement methods for lateral wall thickness, TAD, and the difference in cervical shaft angle

**Fig. 3:**
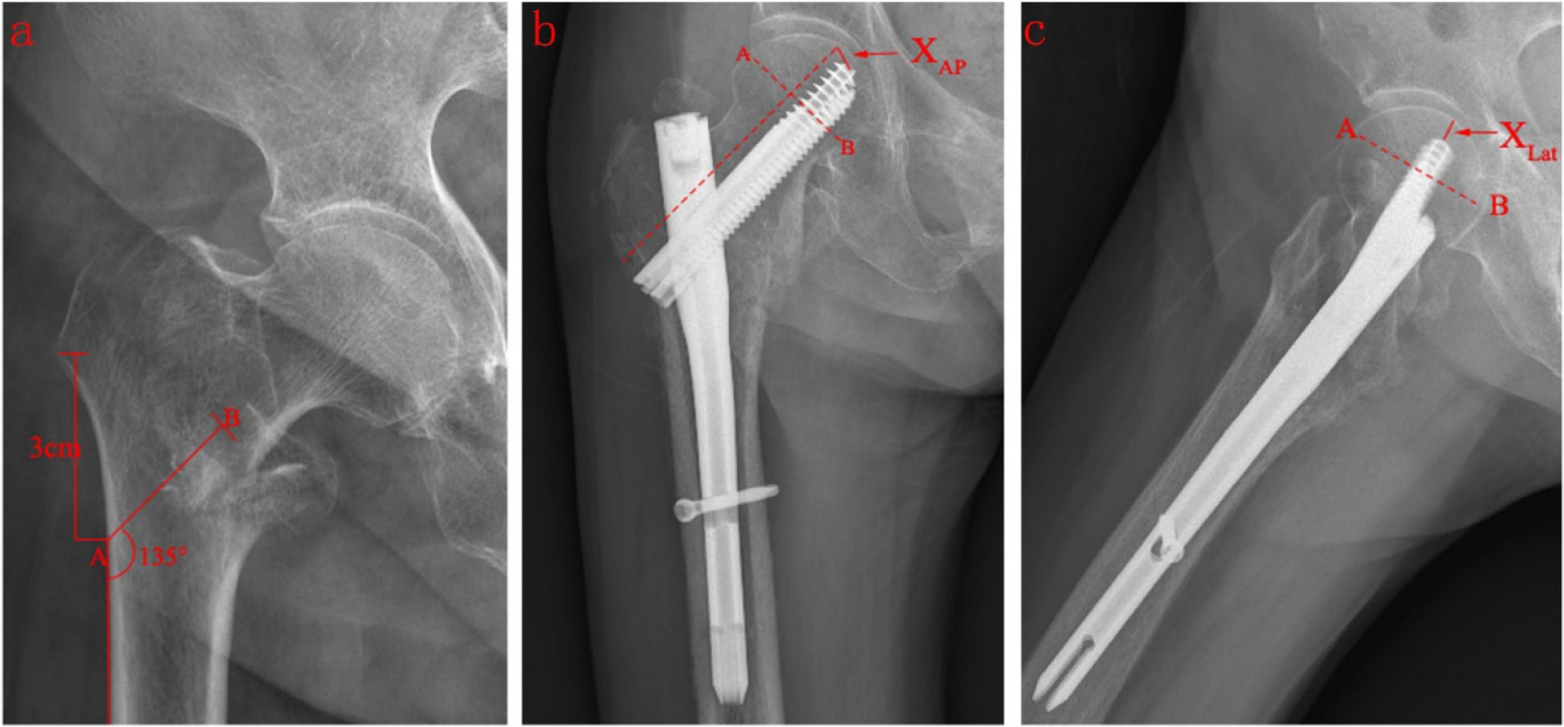
**a** Measurement method for lateral wall thickness. The length of AB is the thickness of the lateral wall. **b** The distance from the tip of the lag screw to the top of the femoral head was measured in the anteroposterior (AP) view. **c** The distance from the tip of the lag screw to the top of the femoral head was measured in the lateral (Lat) view

The implant adopts the design of lag screw and compression screw, and the two screws are combined with interlocking to strengthen the anti-rotation effect of the femoral head. The thickness of the lateral wall was measured on the normal plain film of the proximal femur. It was defined as the distance in mm from a reference point 3 cm below the innominate tubercle of the greater trochanter to the fracture line (the midline between the two cortex lines) at an angle of 135° in the anteroposterior position (Fig. 3a) [13]; 2) the TAD of the InterTAN fixation is the sum of the distances from the tip of the lag screw to the top of the femoral head in the anteroposterior and lateral positions [24] (Fig. 3b, c); and 3) the difference in the neck shaft angle refers to the difference between the neck shaft angle of the contralateral proximal femur and that of the affected side.

### Efficacy evaluation and postoperative management

Visual analog scale (VAS) and Harris scores were recorded 1, 6, 12, 18 and 24 months after the operation. Patient-related functional outcomes were evaluated based on the Harris score (HHS). The HHS was categorized as excellent (90–10), good (79–79), or poor (< 70) [23]. A continuous passive motion (CPM) machine (Smith & Nephew, Shanghai, China) was used to accelerate the recovery of joint function. In order to prevent the formation of deep venous thrombosis (DVT), low molecular weight heparin (60 mg, Shanghai, China) was injected subcutaneously 24 h after the operation for 14 days. Celecoxib (100 mg, Pfizer, USA) was taken orally twice a day for 7 days. According to the patient's situation, opioids were used for analgesia as necessary. On the first day after the operation, anteroposterior films of the bilateral hip joint and the cross table lateral view of the hip were taken to assess the reduction and stability of the fracture. On the first day after the operation, patients could exercise in bed and get out of bed for non-weight-bearing rehabilitation exercises using a walking aid. After 4 weeks, according to the fracture healing and the general condition of the patients, they were encouraged to walk for partial weight-bearing rehabilitation exercises with a walking aid. At 8 weeks postoperatively, patients were encouraged to carry out rehabilitation exercises with complete weight-bearing.

### Statistical analysis

The data were analyzed by SPSS 25.0 (IBM company, Chicago, USA). All measurement data are expressed as the means ± standard deviations and 95% confidence intervals (CIs). Repeated measures analysis of variance was used for comparisons between time points within the same group. The least significant difference (LSD) or Tamhane test was used to compare groups. The Spearman method was used for correlation analysis. Logistic analysis was used to identify the risk factors for internal fixation failure. The Kaplan–Meier test was used to test the internal fixation and patient survival time. The chi-square test was used to analyze the proportion of internal fixation failure types. Inter- and intraobserver reliability was evaluated by intraclass correlation coefficient (ICC) values. The significance level was *P* = 0.05.

## Results

### VAS scores

**Fig. 4:**
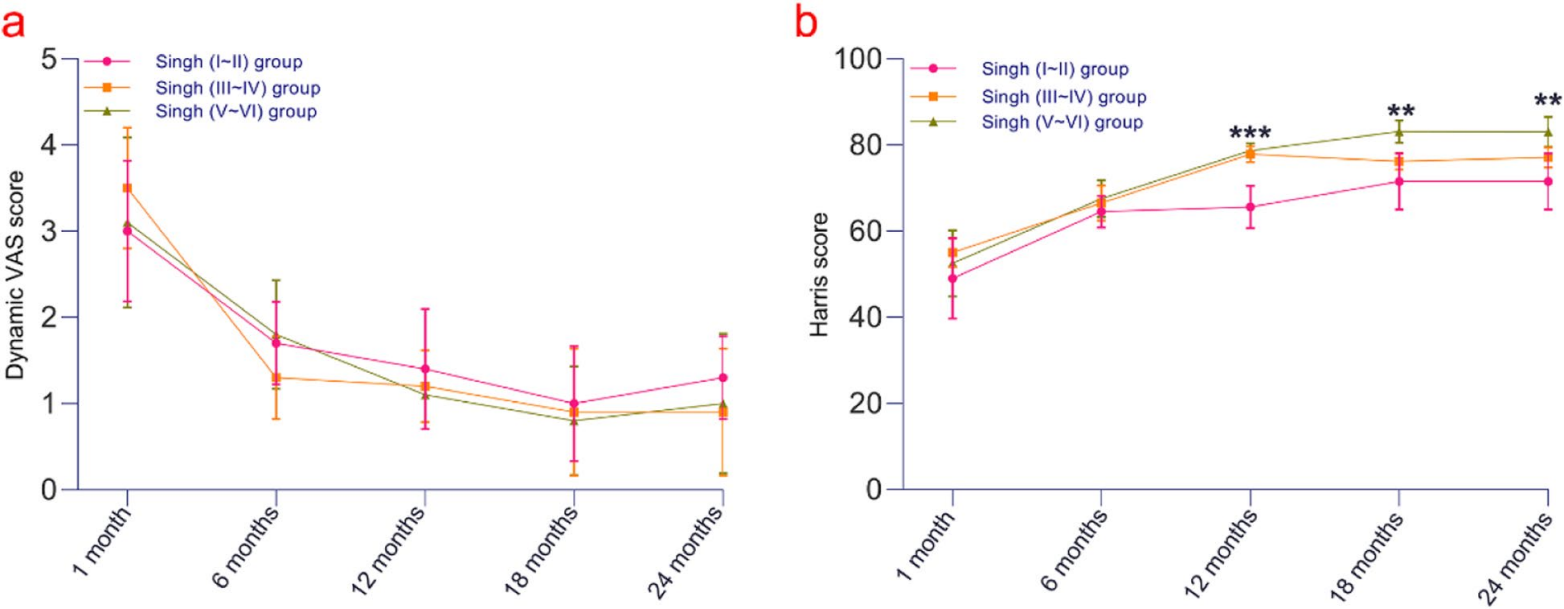
**a** At 6, 12, 18 and 24 months postoperatively, the dynamic VAS scores of the three groups were significantly improved compared with those at 1 month after the operation (*P* = 0.00), and there was no significant difference in the pain scores among the three groups (*P* > 0.05). **b** The Harris scores of the Singh (I ~ II) group were lower than those of the other two groups at 12, 18 and 24 months after surgery (*P* < 0.01)

At 6, 12, 18 and 24 months after surgery, the dynamic VAS scores of the three groups were significantly improved (*P* = 0.00), and from 1 to 24 months after surgery, the VAS scores of the Singh (I ~ II), Singh (III ~ IV) and Singh (V ~ VI) groups ranged from (95% CI, 2.42 to 3.58) to (95% CI, 0.95 to 1.65) with *P* = 0.00, from (95% CI, 2.99 to 4.01) to (95% CI, 0.37 to 1.42) with *P* = 0.00, and from (95% CI, 2.39 to 3.81) to (95% CI, 0.42 to 1.58) with *P* = 0.00 (Fig. 4a). There was no significant difference among the three groups (*P* > 0.05).

### Functional score

At 6, 12, 18 and 24 months after surgery (Fig. 4b), the Harris scores of the three groups were significantly improved (*P* = 0.00). At 12, 18 and 24 months postoperatively, the Harris scores of the Singh (I-II) group were (95% CI, 62.04 to 69.16), (95% CI, 66.80 to 76.20), and (95% CI, 66.80 to 76.20), and hip function was not improved compared with that of the other two groups (*P* < 0.01).

### Correlation analysis

**Fig. 5:**
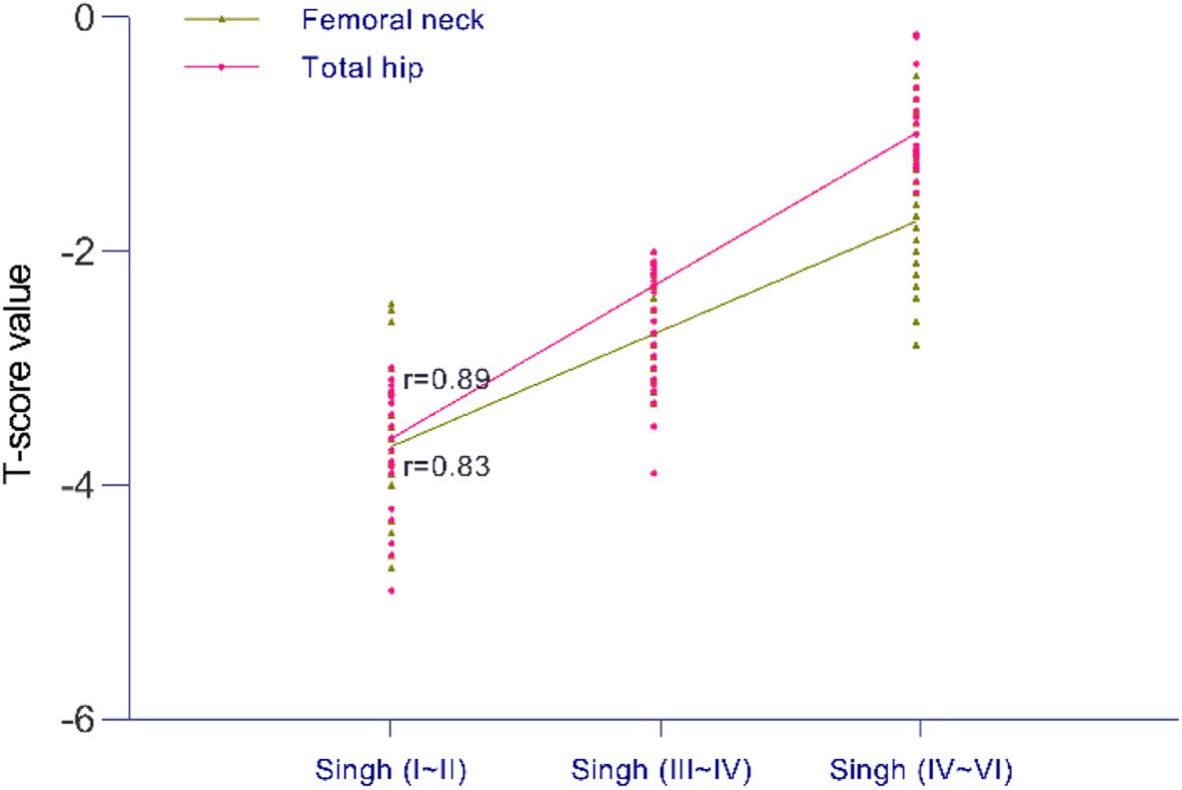
There was a strong correlation between the Singh index and the T-score of the total hip and femoral neck (*P* = 0.00)

The bone mineral density (BMD) of the femoral neck and intertrochanteric region of 40 patients in each group was measured by the dual energy X-ray method. The patients were characterized as either osteoporotic (T score ≥ -2.5 S.D.), osteopenic (T score ≥ -2.5 S.D. and < -1.0 S.D.) or normal (T score > -1.0 S.D.) in terms of BMD. The T-scores of the total hip and femoral neck in the three groups ranged from (95% CI, -3.65 to -3.33) to (95% CI, -1.00 to -0.76) and from (95% CI, -3.84 to -3.49) to (95% CI, -1.92 to -1.56), respectively. Spearman correlation analysis showed that the Singh index had a significant positive correlation with the dual energy X-ray T-score of the femoral neck and total hip (*P* = 0.00, *r* = 0.83; *P* = 0.00, *r* = 0.89); the r value was between 0.8 and 1.0, indicating a very strong correlation (Fig. 5).

### The relationship of the Singh index and internal fixation failure

**Fig. 6:**
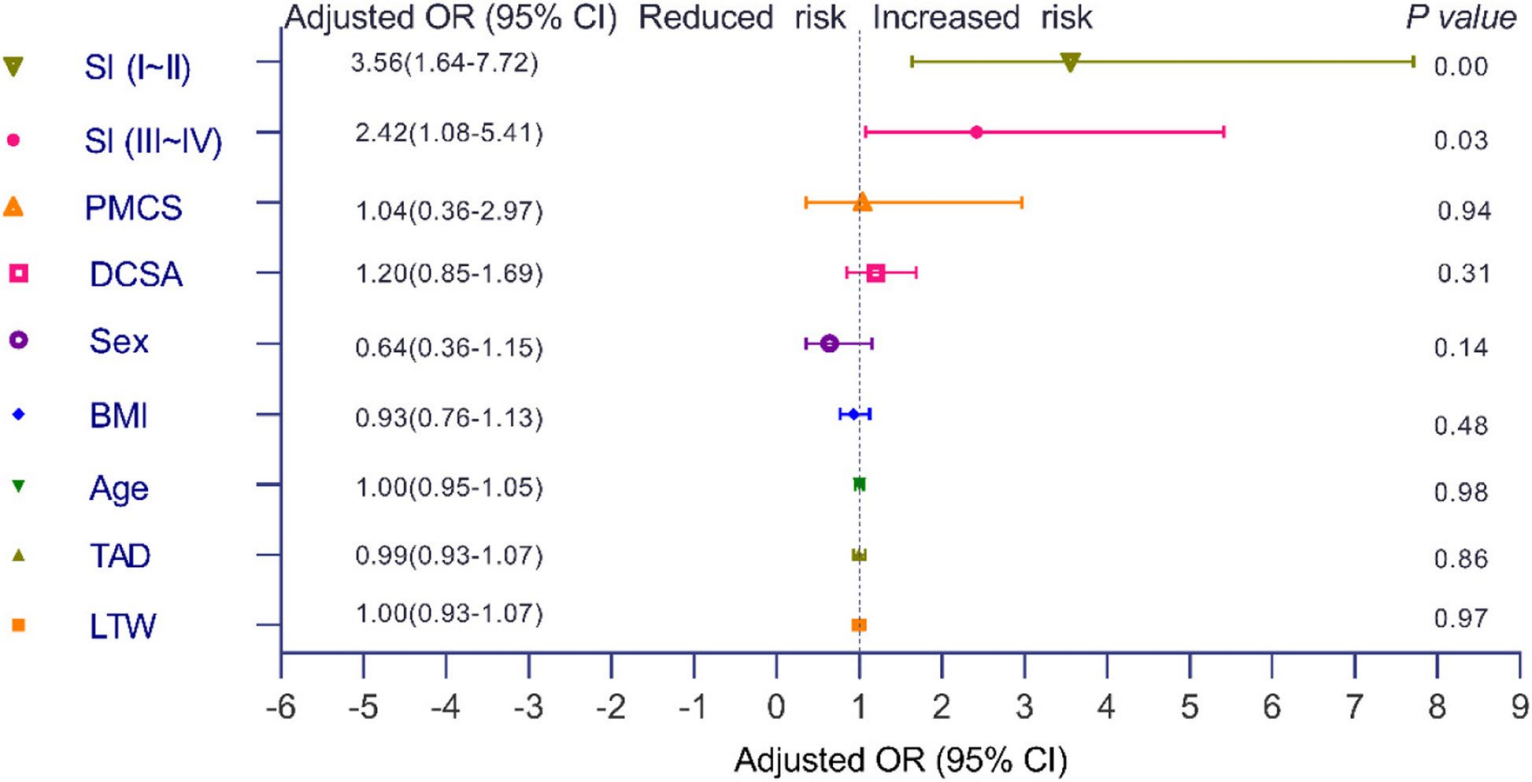
Logistic regression analysis showed that covariates, including PMCS, DCSA, sex, age, BMI, TAD and LWT, had no significant effect on internal fixation failure (*P* > 0.05). After excluding other mixed factors (PMCS: positive medial cortex support; DCSA: difference in cervical shaft angle; BMI: body mass index; TAD: tip-apex distance; LTW: lateral wall thickness), the SI (I ~ II) and SI (III ~ IV) groups had adjusted ORs of 3.56 (95% CI, 1.64 to 7.72) and 2.42 (95% CI, 1.08 to 5.41), respectively, with *P* = 0.00 and *P* = 0.03. The Singh index was an independent factor for predicting the failure of internal fixation. Every decrease in the Singh index can increase the failure rate of internal fixation

The study was conducted to analyze the Singh index (SI). Crude odds ratios (OR) in SI (I ~ II) and SI (III ~ IV) groups = 3.67 (95% CI, 1.70 to 7.09) and 2.33 (95% CI, 1.05 to 5.20) with *P* = 0.00 and *P* = 0.04 were found by logistic regression. After excluding other confounding factors, including positive medial cortex support (PMCS), difference in cervical shaft angle (DCSA), sex, age, BMI, TAD, and lateral wall thickness (LWT), we observed adjusted ORs in the SI (I ~ II) and SI (III ~ IV) groups of 3.56 (95% CI, 1.64 to 7.72) and 2.42 (95% CI, 1.08 to 5.41), respectively, with *P* = 0.00 and *P* = 0.03 (Fig. 6).

### Survival analysis (internal fixation and mortality)

**Fig. 7:**
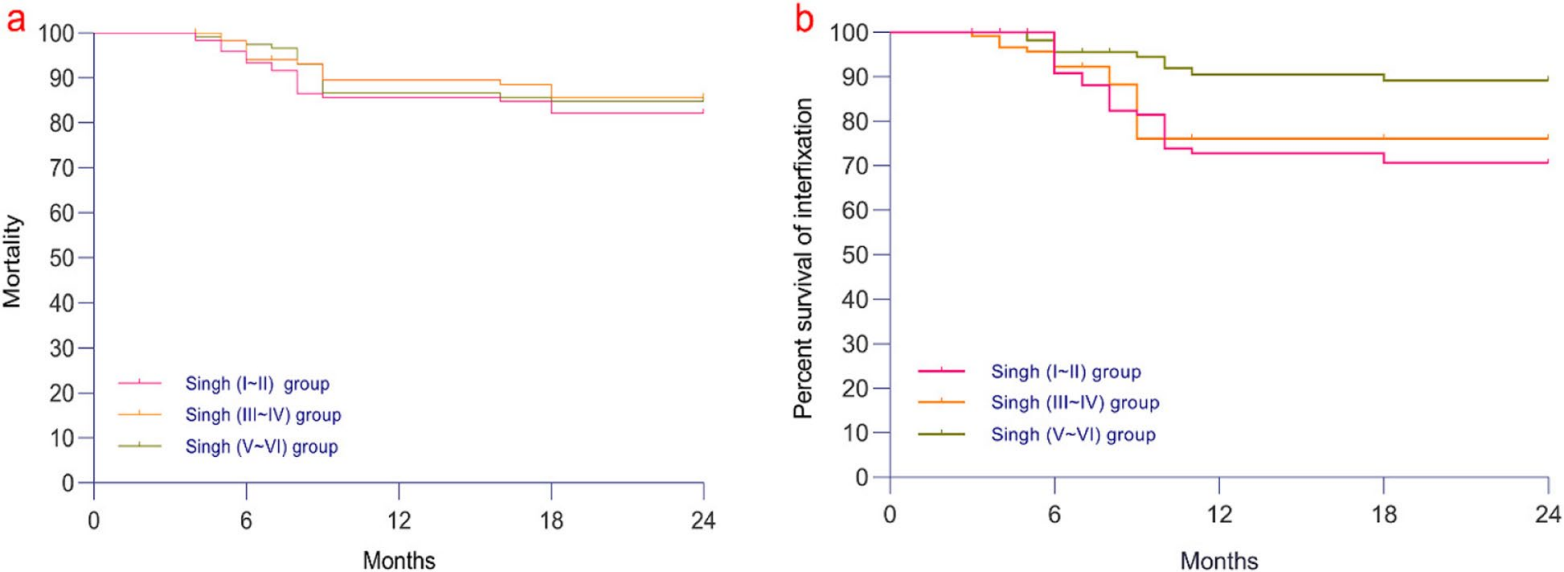
**a** Kaplan–Meier survival analysis showed that there was no significant difference in 24-month mortality among the three groups (*P* = 0.80). **b** Analysis of internal fixation failure in the three groups showed that the 24-month survival rate in the Singh (I ~ II) group was the lowest (*P* = 0.01)

Kaplan–Meier survival analysis (Fig. 7a) showed that there was no significant difference in 24-month mortality among the three groups (*P* = 0.80). Analysis of internal fixation failure in the three groups (Fig. 7b) showed that the 24-month survival rate in the Singh (I ~ II) group was the lowest (*P* = 0.01).

### Main types of internal fixation failure

**Fig. 8:**
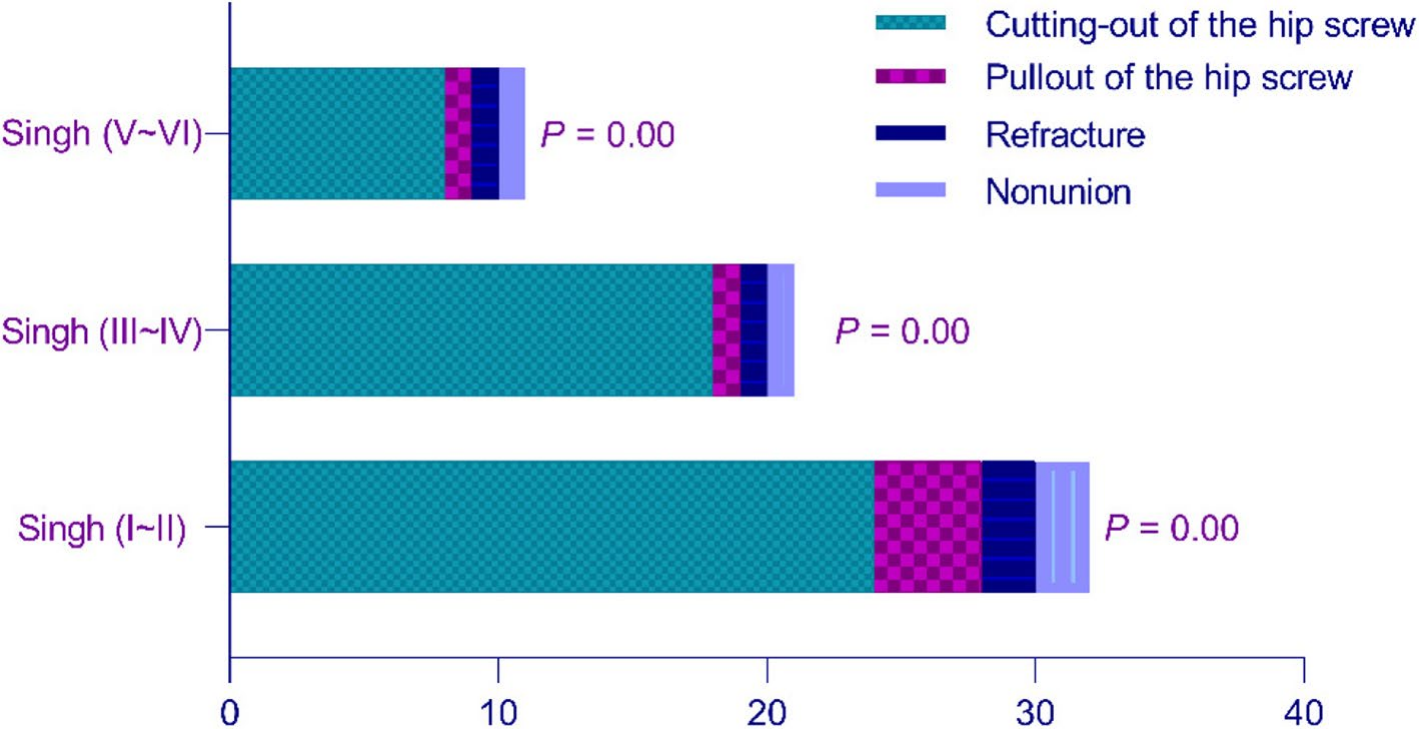
**a**, **b** An 84-year-old woman underwent InterTAN fixation; 9 months postoperatively, cutting-out of the lag screw from the femoral head could be seen on the anteroposterior (AP) view, and the lateral view showed cutting-out of the lag screw. **c** A 90-year-old man who was diagnosed with intertrochanteric fracture underwent InterTAN fixation to fix the fracture; 11 months after the operation, the lag screw pulled out from the bone

**Fig. 9:**
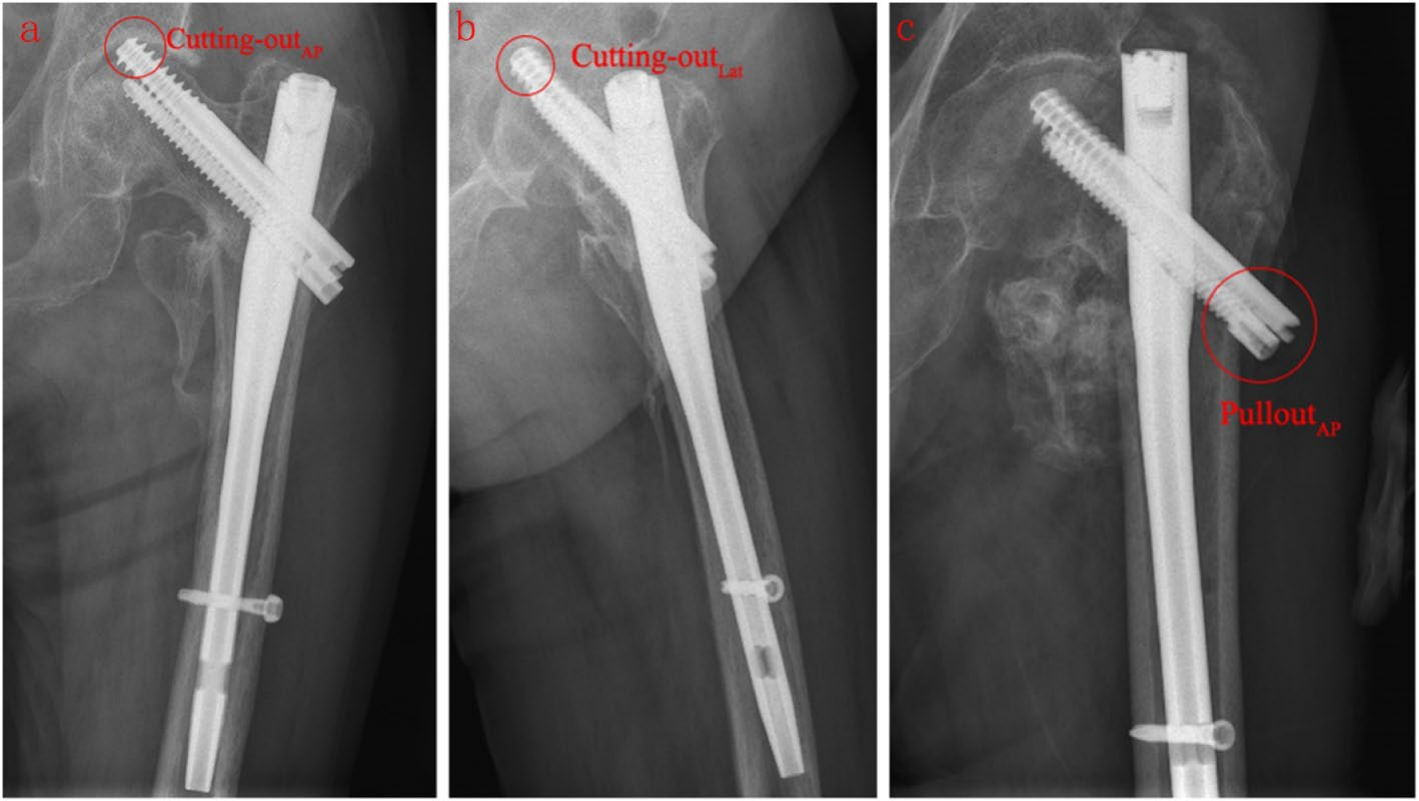
Lag screw cutting-out was the main type of internal fixation failure in the three groups (*P* = 0.00). The incidence of lag screw cutting-out was highest in the Singh (I ~ II) group (*P* = 0.00)

The main types of internal fixation failure in the three groups were analyzed, including cutting-out of the lag screw (Fig. 8a, b), pullout of the lag screw (Fig. 8c), refracture of the affected side and nonunion of the fracture (Fig. 9). The number of cut-outs in the Singh (I ~ II), Singh (III ~ IV), and Singh (V ~ VI) groups was 24, 18, and 8 patients, respectively; the number of pullouts was 4, 1, and 1 patients; the number of refractures was 2, 1, and 1 patients; and the number of nonunions was 2, 1, and 1 patients. The main complications of internal fixation in the three groups was lag screw cutting-out (*P* = 0.00). The incidence of lag screw cutting-out was highest in the Singh (I ~ II) group (*P* = 0.00).

## Discussion

This retrospective study shows that InterTAN fixation is an effective surgical method for the treatment of unstable hip fractures in elderly patients and can effectively improve hip pain and function. The Singh index has a significant correlation with bone mineral density in the femoral neck and total hip. A low Singh index is associated with a reduced InterTAN fixation survival time, which can increase the risk of internal fixation failure. The main type of internal fixation failure is the cutting-out of the lag screw. For patients with a low Singh index, hip joint replacement may be a suitable option. Because most patients who are diagnosed with intertrochanteric fracture need emergency surgery [25–27], the Singh index, as a simple, fast and inexpensive imaging-based evaluation tool, can be a helpful guide for clinicians in the selection of appropriate surgical methods and postoperative interventions for these patients.

This study recorded the VAS and Harris scores of three groups 48 months postoperatively and found that InterTAN fixation is an effective treatment for elderly patients with intertrochanteric fracture and that it can effectively improve hip pain and function, in agreement with previous clinical research results [6, 28, 29]. However, 12 months after the operation, the hip function of the Singh (I~II) group was not as good as that of the other two groups. The main reason may be that the incidence of InterTAN fixation failure in the Singh (I~II) group was high. The main type of fixation failure was hip screw cutting-out. The main treatment method adopted for these patients was to remove the lag screw. The patients needed to be partially weight bearing, which would affect the overall Harris scores of this group.

In this clinical study, Spearman correlation analysis was used to analyze the correlation between the Singh index and the T-score of the total hip and femoral neck in the three groups. The Singh index was highly correlated with the T-score of the femoral neck and total hip. Although previous studies showed no significant correlation between the Singh index and hip bone mineral density, the number of cases in this part of the study was small, and the evidence level was not high for the population in specific areas [30]. The results of this study support the Singh index as a simple and inexpensive imaging-based evaluation method for hip bone mineral density, which is consistent with most clinical research results [31–34].

Kaplan-Meier survival analysis was also used to analyze the fixation and survival time of the patients within 48 months after the InterTAN operation. There was no significant difference in mortality among the patients. The main reason is that the three groups were treated with emergency InterTAN fixation within 24 hours [25, 26, 35]. Regardless of the bone mass of the hip, the patients in the three groups could perform early functional exercise. Pressure sores, pneumonia and deep vein thrombosis were prevented, and the mortality rate was reduced. The results also showed that the rate of internal fixation failure in the Singh (I~II) group was higher than that in the other two groups. The main reason may be that the density and mechanical strength of hip bone in the Singh (I~II) group were weak, which led to insufficient control of the InterTAN device in bone issues, which could easily have led to the occurrence of hip screw cutting-out, nail withdrawal, and refracture.

Although there are many factors that lead to internal fixation failure, the most important factors include age, BMI, thickness of the lateral wall, TAD, and the extent of positive medial wall support. This study excluded the influence of relevant confounding factors. The Singh index was an independent risk factor for internal fixation failure according to logistic regression. As the Singh index increases, the internal fixation failure rate is reduced, which is in line with the finding that the Singh (I~II) group had a shorter internal fixation survival time.

The main types of InterTAN fixation failure are lag screw cutting-out, screw pullout and refracture. This study found that hip screw cutting-out is the main type of postoperative internal fixation failure, which is consistent with the conclusions of most clinical studies [36, 37]. The incidence of lag screw cutting-out in the three groups was significantly higher than that in previous studies [38–40]. An important reason may be that the research subjects were elderly patients in Asia. The loss of trabecular bone density leads to a more significant decrease in bone strength [41, 42], which leads to a decrease in the forces holding the screw in the bone and prohibits InterTAN fixation from fully compressing the fracture. As patients walk in the later stage, the risk of screw cutting-out increases under the dual effects of bone strength decline and fracture fretting. To enhance the forces holding the screws in place and reduce the rate of internal fixation failure for patients with a high risk of lag screw cutting-out, it is suggested that InterTAN fixation should be combined with bone cement fixation, and evidenced-based anti-osteoporosis treatment should be used in patients after surgery.

The limitations of this study are as follows: first, logistic regression analysis was used to analyze the correlation between the Singh index and internal fixation failure. The most important confounding factors were included, but not all confounding factors were excluded. Second, the observation time was short, and a longer follow-up is needed to understand the survival time of the internal fixation and the patients. Third, it is difficult to distinguish adjacent Singh index grades in the clinic. However, the grouping method may be more suitable for clinical work. Fourth, the results obtained can be used for this specific implant but are not transferable to others. For the users of this implant, the Singh index can be used preoperatively to safely identify patients at risk for implant failure. Meanwhile, it can guide orthopedic surgeons to choose more reasonable treatment schemes, such as hip replacement, so that patients can obtain better clinical efficacy. Future studies should investigate the quality of alternative therapies. However, it is not possible to make a generalization on the basis of these data.

## Conclusion

The Singh index has a significant correlation with the bone mineral density of the femoral neck and total hip. The Singh (I ~ II) group had lower Harris scores and a lower internal fixation survival rate. The Singh index can be used as an independent predictor of internal fixation failure after InterTAN fixation, especially the risk of lag screw cutting-out.

## Supplementary Information

**Additional file 1.**

## Data Availability

The datasets used and/or analysed during the current study are available from the corresponding author on reasonable request.

## Abbreviations

PMCS: Positive medial cortex support
DCSA: Difference in cervical shaft angle
BMI: Body mass index
TAD: Tip-apex distance
LTW: Lateral wall thickness

## References

[1] Yuan BJ, Abdel MP, Cross WW, Berry DJ (2017). Hip arthroplasty after surgical treatment of intertrochanteric hip fractures. J Arthroplasty.

[2] Zhang H, Zhu X, Pei G, Zeng X, Zhang N, Xu P (2017). A retrospective analysis of the InterTan nail and proximal femoral nail anti-rotation in the treatment of intertrochanteric fractures in elderly patients with osteoporosis: a minimum follow-up of 3 years. J Orthop Surg Res.

[3] Selim A, Ponugoti N, Naqvi AZ, Magill H (2021). Cephalo-medullary nailing versus dynamic hip screw with trochanteric stabilisation plate for the treatment of unstable per-trochanteric hip fractures: a meta-analysis. J Orthop Surg Res.

[4] Ekinci Y, Gürbüz K, Batın S, Kahraman M, Doğar F, Kaya EZ (2020). A multicenter intertrochanteric fracture study in the elderly: Hemiarthroplasty versus proximal femoral nailing. Jt Dis Relat Surg.

[5] Zhang H, Zeng X, Zhang N, Zeng D, Xu P, Zhang L (2017). INTERTAN nail versus proximal femoral nail antirotation-Asia for intertrochanteric femur fractures in elderly patients with primary osteoporosis. J Int Med Res.

[6] Wang Q, Yang X, He HZ, Dong LJ, Huang DG (2014). Comparative study of InterTAN and Dynamic Hip Screw in treatment of femoral intertrochanteric injury and wound. Int J Clin Exp Med.

[7] Sellan M, Bryant D, Tieszer C, Papp S, Lawendy A, Liew A (2019). Short Versus Long InterTAN Fixation for Geriatric Intertrochanteric Hip Fractures: A Multicentre Head-to-Head Comparison. J Orthop Trauma.

[8] Luo W, Fu X, Ma JX, Huang JM, Wu J, Ma XL (2020). Biomechanical comparison of INTERTAN Nail and gamma3 nail for intertrochanteric fractures. Orthop Surg.

[9] Liu W, Liu J, Ji G (2020). Comparison of clinical outcomes with proximal femoral nail anti-rotation versus InterTAN nail for intertrochanteric femoral fractures: a meta-analysis. J Orthop Surg Res.

[10] Matre K, Vinje T, Havelin LI, Gjertsen JE, Furnes O, Espehaug B (2013). TRIGEN INTERTAN intramedullary nail versus sliding hip screw: a prospective, randomized multicenter study on pain, function, and complications in 684 patients with an intertrochanteric or subtrochanteric fracture and one year of follow-up. J Bone Joint Surg Am.

[11] Siegel RS (1996). The value of the tip-apex distance in predicting failure of fixation of peritrochanteric fractures of the hip. J Bone Joint Surg Am.

[12] Lobo-Escolar A, Joven E, Iglesias D, Herrera A (2010). Predictive factors for cutting-out in femoral intramedullary nailing. Injury.

[13] Hsu  CE, Shih  CM, Wang  CC, Huang  KC (2013). Lateral femoral wall thickness. A reliable predictor of post-operative lateral wall fracture in intertrochanteric fractures. Bone Joint J.

[14] Raghuraman R, Kam JW, Chua D (2019). Predictors of failure following fixation of intertrochanteric fractures with proximal femoral nail antirotation. Singapore Med J.

[15] Palm H, Jacobsen S, Sonne-Holm S, Gebuhr  P, Hip Fracture Study Group (2007). Integrity of the lateral femoral wall in intertrochanteric hip fractures: an important predictor of a reoperation. J Bone Joint Surg Am.

[16] Chang SM, Zhang YQ, Ma Z, Li Q, Dargel J, Eysel P (2015). Fracture reduction with positive medial cortical support: a key element in stability reconstruction for the unstable pertrochanteric hip fractures. Arch Orthop Trauma Surg.

[17] Singh M, Nagrath AR, Maini PS (1970). Changes in trabecular pattern of the upper end of the femur as an index of osteoporosis. J Bone Joint Surg Am.

[18] D'Amelio P, Rossi P, Isaia G, Lollino N, Castoldi F, Girardo M (2008). Bone mineral density and singh index predict bone mechanical properties of human femur. Connect Tissue Res.

[19] Liu Z, Gao H, Bai X, Zhao L, Li Y, Wang B (2017). Evaluation of singh index and osteoporosis self-assessment tool for asians as risk assessment tools of hip fracture in patients with type 2 diabetes mellitus. J Orthop Surg Res.

[20] Cristofolini L (2007). Fractures of the proximal femur: correlates of radiological evidence of osteoporosis. Skeletal Radiol.

[21] Pellegrini A, Tacci F, Leigheb M, Costantino C, Pedrazzini A, Pedrazzi G (2017). Injuries of the trochanteric region: can analysis of radiographic indices help in prediction of recurrent osteoporotic hip fractures. Acta Biomed.

[22] Aaron JE, Shore PA, Shore RC, Beneton M, Kanis JA (2000). Trabecular architecture in women and men of similar bone mass with and without vertebral fracture: II. Three-dimensional histology Bone.

[23] Hauschild O, Ghanem N, Oberst M, Baumann T, Kreuz PC, Langer M (2009). Evaluation of Singh index for assessment of osteoporosis using digital radiography. Eur J Radiol.

[24] Kaynak G, Ünlü MC, Güven MF, Erdal OA, Tok O, Botanlıoğlu H (2018). Intramedullary nail with integrated cephalocervical screws in the intertrochanteric fractures treatment: Position of screws in fracture stability. Ulus Travma Acil Cerrahi Derg.

[25] Novack V, Jotkowitz A, Etzion O, Porath A (2007). Does delay in surgery after hip fracture lead to worse outcomes? A multicenter survey. Int J Qual Health Care.

[26] Delaveau A, Saint-Genez F, Gayet LE, Paccalin M, Ounajim A, Vendeuvre T (2019). Impact of time to surgery in upper femoral fracture in orthogeriatrics. Orthop Traumatol Surg Res.

[27] Ogawa T, Aoki T, Shirasawa S (2019). Effect of hip fracture surgery within 24 hours on short-term mobility. J Orthop Sci.

[28] Seyhan M, Turkmen I, Unay K, Ozkut AT (2015). Do PFNA devices and Intertan nails both have the same effects in the treatment of trochanteric fractures? A prospective clinical study. J Orthop Sci.

[29] Huang Y, Zhang C, Luo Y (2013). A comparative biomechanical study of proximal femoral nail (InterTAN) and proximal femoral nail antirotation for intertrochanteric fractures. Int Orthop.

[30] Soontrapa S, Soontrapa S, Srinakarin J, Chowchuen P (2005). Singh index screening for femoral neck osteoporosis. J Med Assoc Thai.

[31] Karlsson KM, Sernbo I, Obrant KJ, Redlund-Johnell I, Johnell O (1996). Femoral neck geometry and radiographic signs of osteoporosis as predictors of hip fracture. Bone.

[32] Vaseenon T, Luevitoonvechkij S, Namwongphrom S, Rojanasthien S. Proximal femoral bone geometry in osteoporotic hip fractures in Thailand. J Med Assoc Thai. 2015;98:77–81. 25775736

[33] Masud T, Jawed S, Doyle DV, Spector TD (1995). A population study of the screening potential of assessment of trabecular pattern of the femoral neck (Singh index): the Chingford Study. Br J Radiol.

[34] Patel SH, Murphy KP (2006). Fractures of the proximal femur: correlates of radiological evidence of osteoporosis. Skeletal Radiol.

[35] Simunovic N, Devereaux PJ, Sprague S, Guyatt GH, Schemitsch E, Debeer J (2010). Effect of early surgery after hip fracture on mortality and complications: systematic review and meta-analysis. CMAJ.

[36] Yoo J, Chang J, Park C, Hwang J (2020). Risk Factors Associated with Failure of Cephalomedullary Nail Fixation in the Treatment of Trochanteric Hip Fractures. Clin Orthop Surg.

[37] John B, Sharma A, Mahajan A, Pandey R (2019). Tip-apex distance and other predictors of outcome in cephalomedullary nailing of unstable trochanteric fractures. J Clin Orthop Trauma.

[38] Puthezhath K, Jayaprakash C (2017). Is calcar referenced tip-apex distance a better predicting factor for cutting out in biaxial cephalomedullary nails than tip-apex distance. J Orthop Surg (Hong Kong).

[39] Lopes-Coutinho L, Dias-Carvalho A, Esteves N, Sousa R (2020). raditional distance "tip-apex" vs. new calcar referenced "tip-apex" - which one is the best peritrochanteric osteosynthesis failure predictor. Injury.

[40] Akan K, Cift H, Ozkan K, Eceviz E, Tasyikan L, Eren A (2011). Effect of osteoporosis on clinical outcomes in intertrochanteric hip fractures treated with a proximal femoral nail. J Int Med Res.

[41] Chen P, Li Z, Hu Y (2016). Prevalence of osteoporosis in China: a meta-analysis and systematic review. BMC Public Health.

[42] Babhulkar S (2017). Unstable trochanteric fractures: Issues and avoiding pitfalls. Injury.

