# Supplementary figures and images for: The predictive value of the Singh index for the risk of InterTAN intramedullary fixation failure in elderly patients with intertrochanteric fractures
Source: BMC Musculoskelet Disord. 2022 Aug 12;23:769. doi: 10.1186/s12891-022-05741-8 (PMC9373533; doi:10.1186/s12891-022-05741-8)

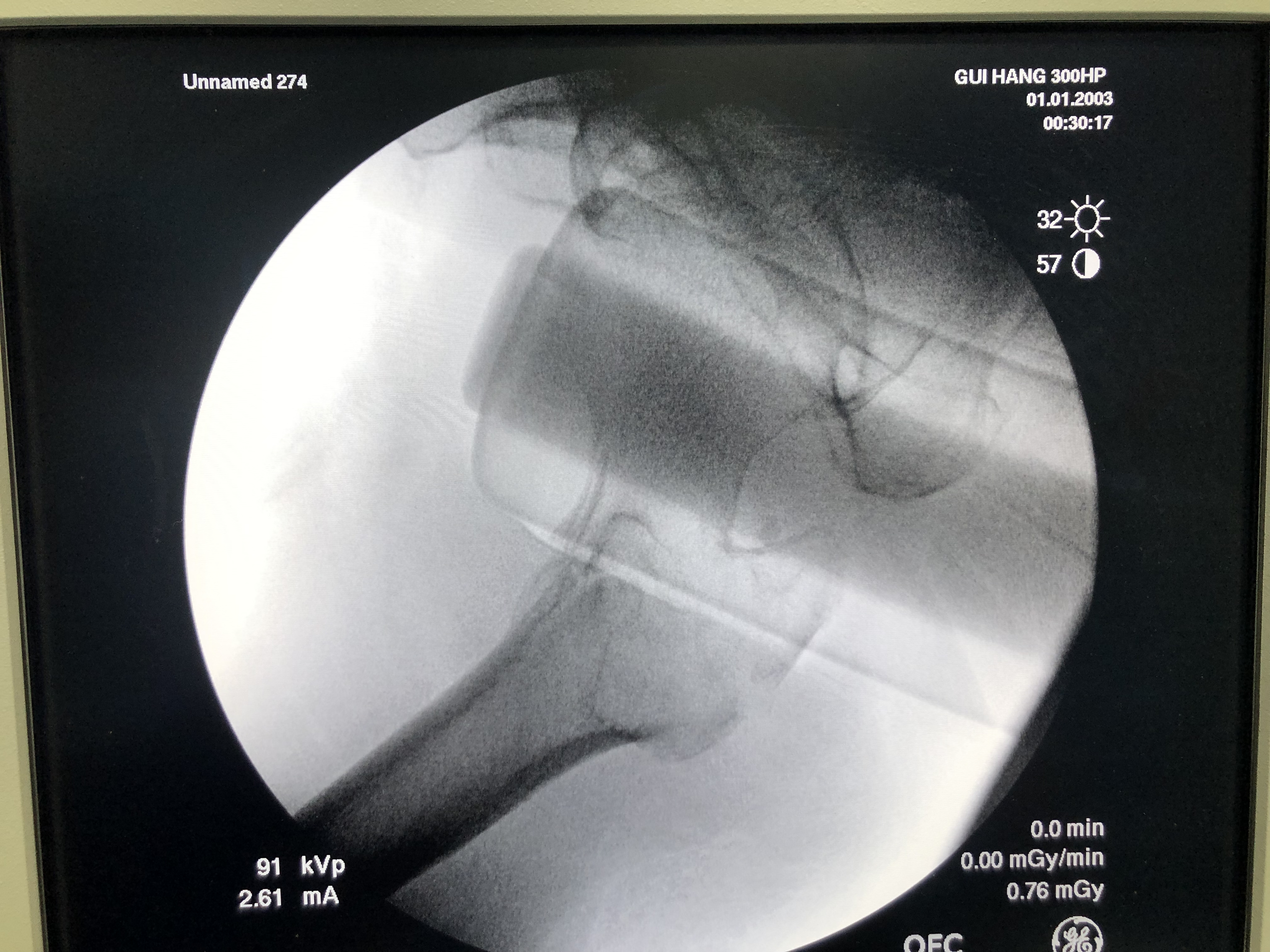

Supplement: Supplementary file 1 — Additional file 1. [file 12891_2022_5741_MOESM1_ESM.zip › Supplementary information/1.jpg]

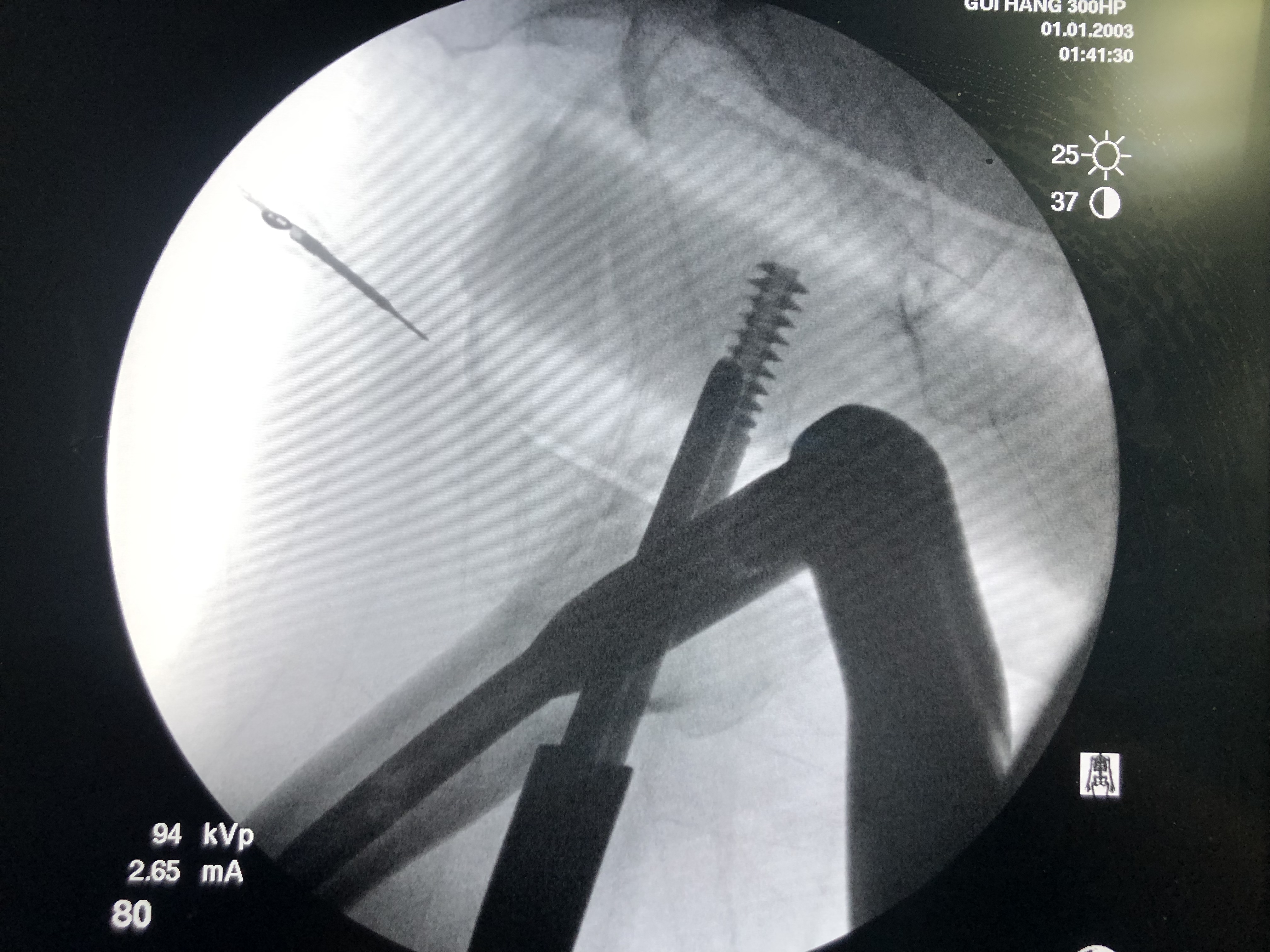

Supplement: Supplementary file 1 — Additional file 1. [file 12891_2022_5741_MOESM1_ESM.zip › Supplementary information/2.jpg]

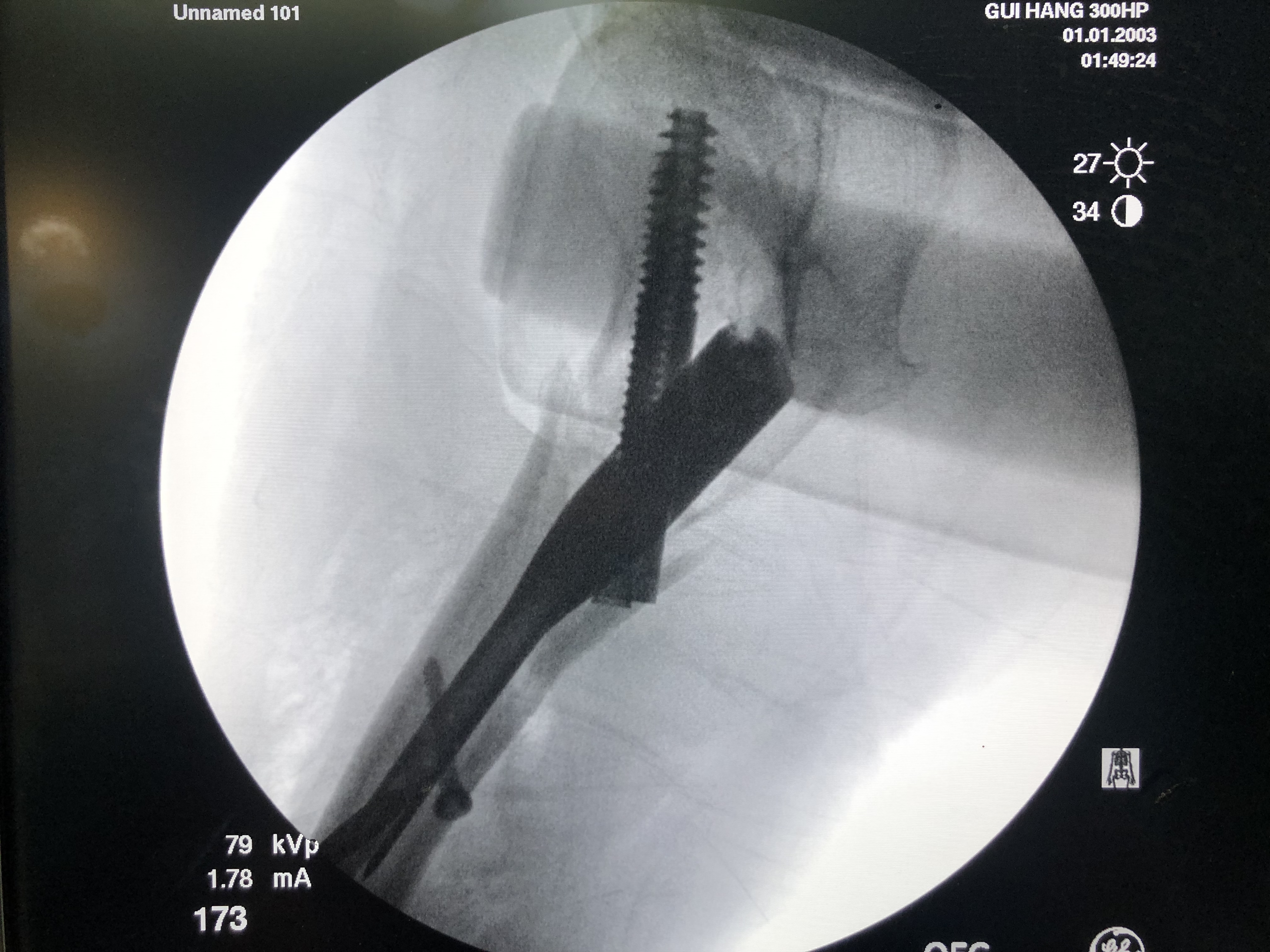

Supplement: Supplementary file 1 — Additional file 1. [file 12891_2022_5741_MOESM1_ESM.zip › Supplementary information/3.jpg]

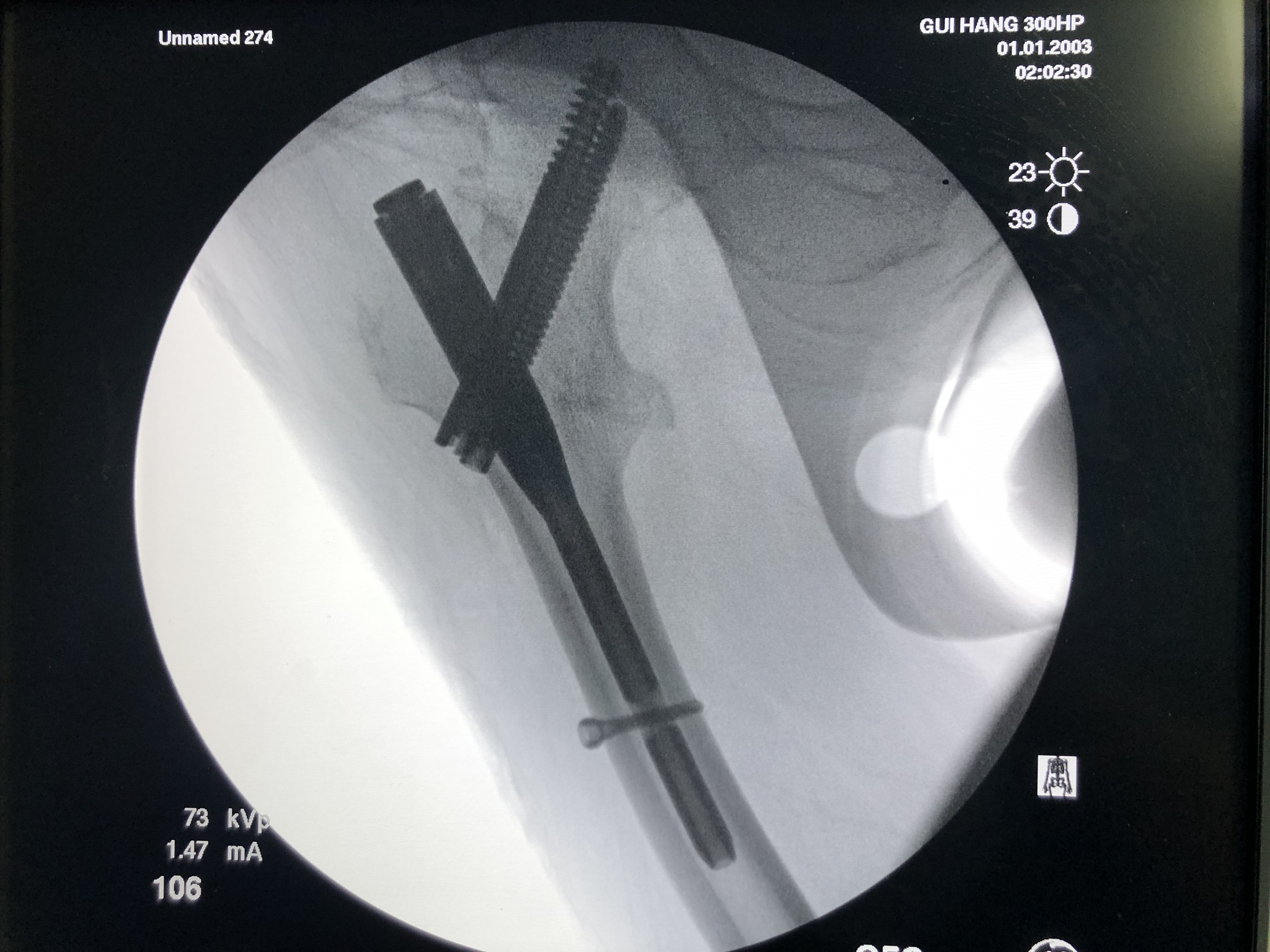

Supplement: Supplementary file 1 — Additional file 1. [file 12891_2022_5741_MOESM1_ESM.zip › Supplementary information/4.jpg]

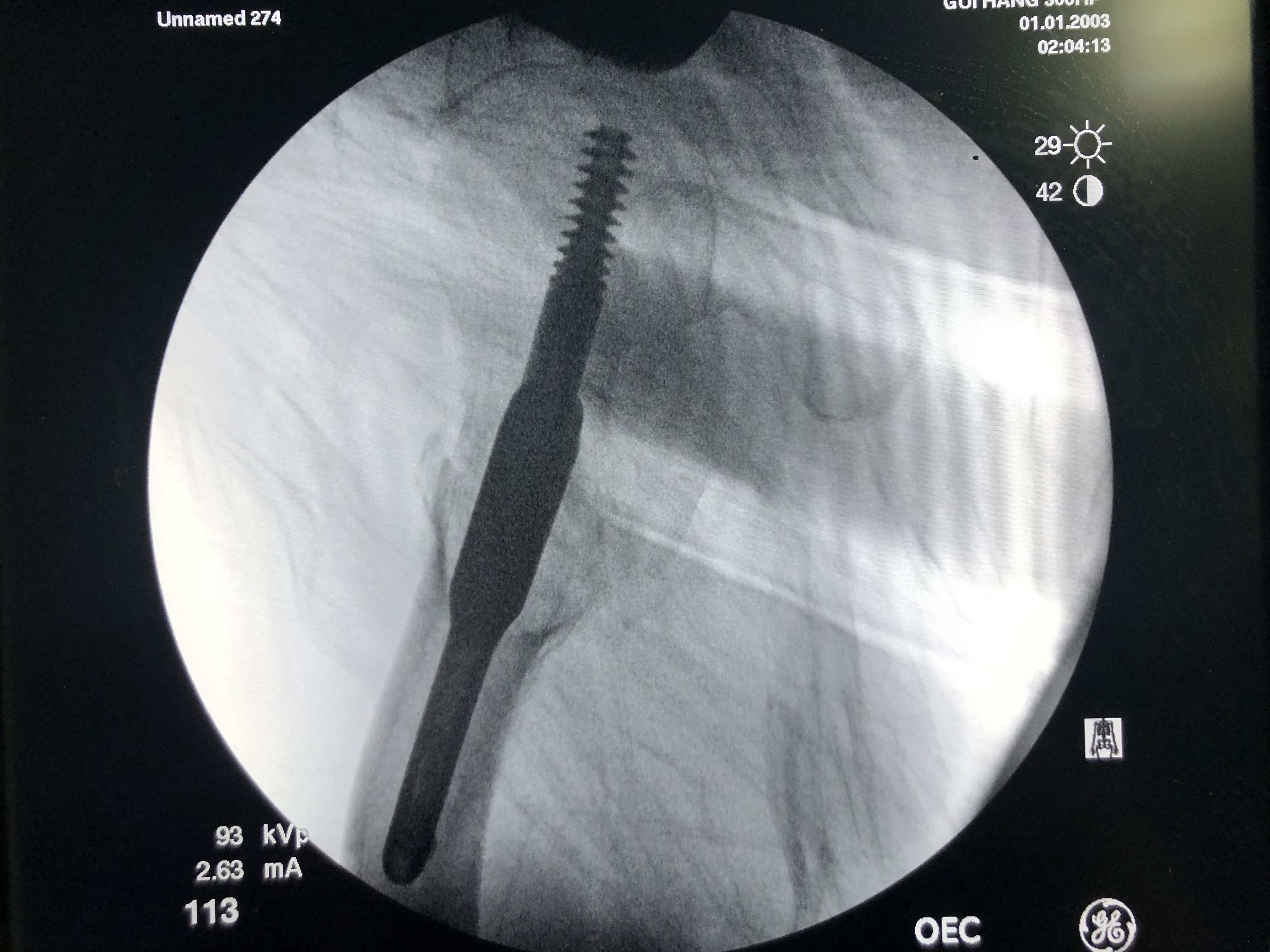

Supplement: Supplementary file 1 — Additional file 1. [file 12891_2022_5741_MOESM1_ESM.zip › Supplementary information/5.jpg]
